# Reachability and the sense of embodiment in amputees using prostheses

**DOI:** 10.1038/s41598-017-05094-6

**Published:** 2017-07-10

**Authors:** Adrienne Gouzien, Fréderique de Vignemont, Amélie Touillet, Noël Martinet, Jozina De Graaf, Nathanaël Jarrassé, Agnès Roby-Brami

**Affiliations:** 1 Institute of Intelligent Systems and Robotics (ISIR), Sorbonne University, UPMC Univ Paris 06, CNRS, UMR 7222, INSERM U1150, Paris, France; 2 Institut Jean Nicod, ENS – EHESS – CNRS UMR 8129, IEC, PSL, Paris, France; 3 Louis Pierquin Centre of the Regional Institute of Rehabilitation, UGECAM Nord-est, Nancy, France; 4 Institute of Movement Sciences (ISM), UMR 7287 - CNRS & Aix-Marseille University, Marseille, France; 5 Service de psychiatrie, Pôle Paris Centre, Hôpitaux de Saint-Maurice, Saint-Maurice, France; 6 Service de psychiatrie, Pôle Paris Centre, Hôpitaux de Saint-Maurice, Saint-Maurice, France

## Abstract

Amputated patients are hardly satisfied with upper limb prostheses, and tend to favour the use of their contralateral arm to partially compensate their disability. This may seem surprising in light of recent evidences that external objects (rubber hand or tool) can easily be embodied, namely incorporated in the body representation. We investigated both implicit body representations (by evaluating the peripersonal space using a reachability judgement task) and the quality of bodily integration of the patient’s prosthesis (assessed via questionnaires). As expected, the patients estimated that they could reach further while wearing their prosthesis, showing an embodiment of their prosthesis in their judgement. Yet, the real reaching space was found to be smaller with their prosthesis than with their healthy limb, showing a large error between reachability judgement and actual capacity. An overestimation was also found on the healthy side (comparatively to healthy subjects) suggesting a bilateral modification of body representation in amputated patients. Finally, a correlation was found between the quality of integration of the prosthesis and the way the body representation changed. This study therefore illustrates the multifaceted nature of the phenomenon of prosthesis integration, which involves its incorporation as a tool, but also various specific subjective aspects.

## Introduction

Even though the psychological and emotional experience of wearing a prosthesis is different for every patient, very few use theirs all day long (25%), most of them only when they need it for a particular activity (50%), and around 25% abandon it after a time^1, 2^. However, the loss of a limb is a major handicap for those patients who are mainly young active people, and technological advances keep on making prostheses more and more efficient to compensate for this disability. One hypothesis to explain why their use remains limited is that, more than the specific technological issues described by patients (weight, battery failure, discomfort on the residual stump, etc.), the problem is the sensorimotor challenge to embody a prosthetic limb.

One can define the notion of embodiment at two distinct levels^3^. At the implicit level of body representations, an object is said to be embodied if some of its properties – or all of them – are processed in the same way as the properties of biological body parts. Embodiment can also be associated with a large range of subjective explicit feelings, including feelings of bodily ownership, feelings of bodily control, of bodily integrity, affective feelings, and so forth. Both implicit and explicit levels of embodiment for extraneous objects have been extensively discussed in the context of the Rubber Hand Illusion in healthy participants. In the classic experimental set up, one sits with one’s arm hidden behind a screen, while fixating on a rubber hand presented in one’s bodily alignment; the rubber hand can then be touched either in synchrony or in asynchrony with one’s hand. In brief, in the synchronous condition participants mislocalize their hand in the direction of the location of the rubber hand and report that it seems as if the rubber hand was their own^4^. A similar illusion can be induced by visuomotor congruency^5^. Tool use is the other main paradigm to assess the malleability of body representations. It has been shown that using a tool quickly modifies (i) the perception of the space surrounding the body (also known as peripersonal space), (ii) the kinematic of subsequent bodily movements and (iii) the perceived size of the limb^6, 7^. These modifications can even occur in hemiplegic patients when the tool is hold by the examiner’arm in a position allowing its embodiment by the patients^8^. Yet, little is knownabout the subjective dimension of tool embodiment.

Prostheses can be conceived as tools insofar as they are a technical medium between the body and the environment. However, they are peculiar tools because they look like bodily parts and their aim is to replace the missing limb instead of extending an existing one. The crucial question is whether these differences make their embodiment easier or more difficult. On the one hand, Miller *et al*.^9^ found that morphological similarity between a tool and an effector increased its embodiment at the implicit level^9^. On the other hand, it has been shown that subjective feelings of embodiment could be induced for non-bodily shaped objects^10^. Here we thus analysed the embodiment of prostheses in amputees, at both implicit and explicit levels.

Patients and the rehabilitation staff do not use the term of embodiment but talk about the objective of “integration” of the prosthesis which is defined very pragmatically as the three degree of compensation for the disability (functional, aesthetic and psychological) with a restoration of autonomy^11, 12^. To our knowledge, no scale has been developed to evaluate this multifactorial integration. We propose here the first scale to operationalise this clinical notion.

At the implicit level, it has been shown that amputees perceived their arm as longer when wearing their prosthesis^13^. Furthermore, a recent study has investigated the effect of prostheses on peripersonal space^14^. Peripersonal space is encoded in a body part-centred frame of reference. As Graziano and Gross (1993, p. 107) described it, it is like “a gelatinous medium surrounding the body that deforms whenever the head rotates or the limbs move”^15^. It is anchored in specific parts of the body and when the body parts move, what is represented as peripersonal space also moves. Numerous studies in monkeys and humans, in both healthy and pathological conditions, have explored the functional features of this specific area close to the body (for a review, see ref. 16). It was after they had found bimodal neurons activated both by tactile stimuli on the skin and by visual stimuli presented in the space *close to the body* of a monkey that Rizzolatti and his colleagues^17^ first introduced the term of peripersonal space. In humans, it has been found that visual stimuli that are presented within peripersonal space can interfere with tactile processing^18^. Such visuo-tactile congruency effects can occur only in peripersonal space because it is only in this space that both visual and tactile experiences share a common spatial frame of reference centred on body parts^19^. A similar effect can be found in the neuropsychological syndrome of tactile extinction. After right-hemisphere lesions, some patients have no difficulty in processing an isolated tactile stimulus on the left side of their body. However, when they are simultaneously touched on the right hand, they are no longer aware of the touch on their left hand. Interestingly, the same is true when they see a visual stimulus near their right hand: the visual stimulus on the right side ‘extinguishes’ the tactile stimulus on the left side so that they fail to detect the touch^20^. Similar multisensory effects have been found with auditory stimuli^21^.

Interestingly, the estimation of peripersonal space stretches and shrinks as the estimated size of the body part stretches and shrinks^14, 22–24^. In a seminal study Iriki and colleagues^6^ trained monkeys to use a rake to reach food placed outside their reaching space. They found that some neurons, which displayed no visual response to food at this far location before training, began to display visual responses after training. In humans, it has been shown that the use of a rake during a few minutes extend the peripersonal space around the hand holding it^25^ and modifies the perceived size of the holding hand which is perceived as longer^26^. A few minutes after tool use was interrupted, the visual receptive fields shrank back to their original size. Likewise, Canzoneri and coll^14^. found that, in patients with upper-limb amputation, the limits of peripersonal space are equally extended by the use of a prosthesis. The authors used an audio-tactile congruency task and found that the amputation had caused a retraction of the peripersonal space around the stump and that the wearing of a prosthesis restored the limits of this space^14^. Thus, peripersonal space surroundingbodily space can provide an implicit access to the embodiment of prostheses.

The cross-modal congruency effect used by Canzoneri and coll., however, focuses exclusively on the sensory dimension of peripersonal space, leaving aside its praxic dimension^27^. Peripersonal space is indeed also the space “within which it [the body] can act”^28^ and the function of tools, prostheses including, is primarily to extend one’s motor abilities. We thus used here a motor task instead of a sensory one to assess the embodiment of prostheses in amputees. As used in previous studies^29^ subjects were asked to judge if they could reach targets presented to them^29^. This reachabiity judgement task requires visually estimating the distance of the target, exploiting bodily knowledge about the functional length of the arm and motor knowledge about the feasibility of the action in the specific context^30–32^. A recent study showed that tool use increased the distance that subjects estimated they could reach^33^. Our study is the first to use the reachability judgement task in amputees to study how amputation and the wearing of a prosthesis affects the representation of the action capacity of both amputated and healthy arms in comparaison to an objective measure of their reaching distance. It is also the first to investigate the relationship between the reachability judgment, which we take as an evidence of the embodiment of the prosthesis and the subjective quality of the integration of the prosthesis assessed from a clinical point of view. To do so, we developed a multi-factor scale based on a questionnaire to evaluate the phenomenology of the prosthesis integration. Twelve patients with hand amputation (at the level of the forearm) participated and the data of eleven of them could be analysed (see Table 1, and Methods for more explanation). Thirty healthy subjects participated as controls. We hypothesized that the subjective quality of the integration of the prosthesis would correlate with the patients’ perception of the limits of their PPS.

**Table 1:** Clinical characteristics of the patients.

	Gender	Age	Dominant hand	Years since amputation	Side of amputation	Level of amputation
P1	M	38	Right	4.88	Right	sup 1/3
P2	M	44	Right	14.12	Right	sup 1/3
*P3*	*M*	*43*	*Left*	*43.67*	*Right*	*agenesis*
P4	F	22	Right	2.49	Left	sup 1/3
P5	M	22	Right	0.13	Right	inf1/3
P6	M	48	Right	10.47	Right	sup 1/3
P7	M	42	Right	2.76	Right	inf1/3
P8	M	44	Right	3.63	Right	RUD
P9	M	61	Right	10.61	Left	sup 1/3
P10	M	50	Right	1.87	Right	middle 1/3
P11	M	29	Right	8.57	Left	sup 1/3
P12	F	51	Left	0.75	Left	sup 1/3

P = patient, M = male, F = female, sup 1/3 = superior third of forearm, inf 1/3 = inferior third of forearm, RUD = Radio-ulnar disarticulation. Healthy subjects – mean age: 41.4 years (min = 23–max = 68), Sex ratio M/F: 5.Patient P3 couldn’t be analysed properly because of “out of range” overestimations within the experiments.

## Results

### Evaluation of the actual action space

In order to evaluate the actual distance that the patients could reach, we measured the maximum reachable distance (MRD) of each patient’s healthy and prosthetic arm (i.e., the amputated limb wearing the prosthesis). The patients were instructed to touch the furthest point on the table with the tested limb. Their trunk was placed in contact with the table while their chin was positioned on a rest in order to limit possible trunk and shoulder movements (see Methods for more information).

**Figure 1:**
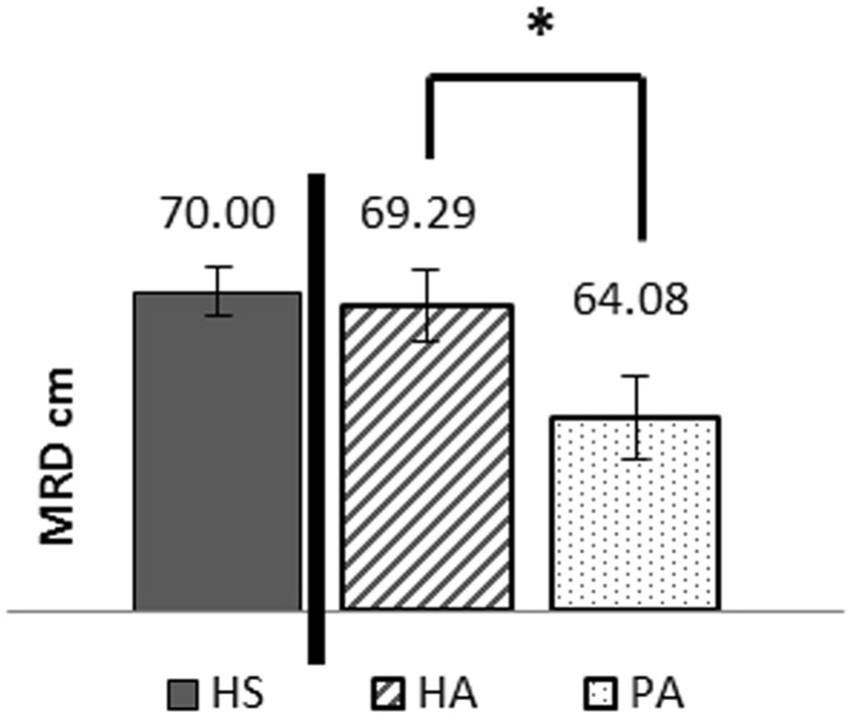
Maximal reachable distance (MRD) in cm for each condition HS = Healthy Subject, HA = Healthy Arm of Amputated Patient, PA = Prosthetic Arm of Amputated Patient *indicate a significant difference between the two groups of participants with p< *0.05*.

A paired t-test with a Bonferroni correction was used and showed that there was a significant difference between the MRD of the healthy arm (69.29 ± 5.82 cm) and the MRD of the prosthetic arm (64.08 ± 6.71 cm; p < 0.01), showing that the patients could reach further with their healthy arm than with their prosthetic arm (Fig. 1).

This difference did not appear to be related to the limb length sincea Student paired t-test showedno significant difference between the length of the two limbs (healthy arm = 76.58 ± 5.88 cm, prosthetic arm = 77.00 ± 5.79 cm; p > 0.05). However, Bayesian analysis showed anecdotal evidence for the null hypothesis (Bayes factor BF_01_ = 1.449) indicating that more information would be needed to conclude to the same size of the two limbs.

The MRD of the healthy subjects (70.00 ± 6.23 cm) and the healthy limb of the patients (69.29 cm ± 5.82) were not significantly different at the Student’s unpaired t test. Once again, Bayes factor showed anecdotal evidence for the null hypothesis (BF_01_ = 2.919), inviting to interpret this result with reserve.

### Evaluation of the limits of the action space determined by the subject

In order to evaluate the effects of wearing a prosthesis on the subjective feeling of capacity of action, each patient underwent a reachability judgement task with both his/her healthy and prosthetic arm. For each trial, the subject was asked to judge if he/she could reach a target projected on the table (covered with a black projection screen) in front of which he was seated. The healthy subjects carried out the same task. For each limb, the target distance was calculated as a function of the MRD measured at the beginning of the experiment. Each of the 25 possible target positions (covering a distance from +9.6 to −9.6 cm from the MRD in steps of 8 mm) was proposed 5 times in a randomised order (total of 125 trials). The responses “Yes or No” obtained in the three conditions (healthy subjects, healthy and prosthetic arms of the patients) were studied with a logistic regression for each individual participant in each condition in order to quantify the limit of the distance they judged as reachable (RJ = Reachability Judgement) (see Methods for more information).

**Figure 2:**
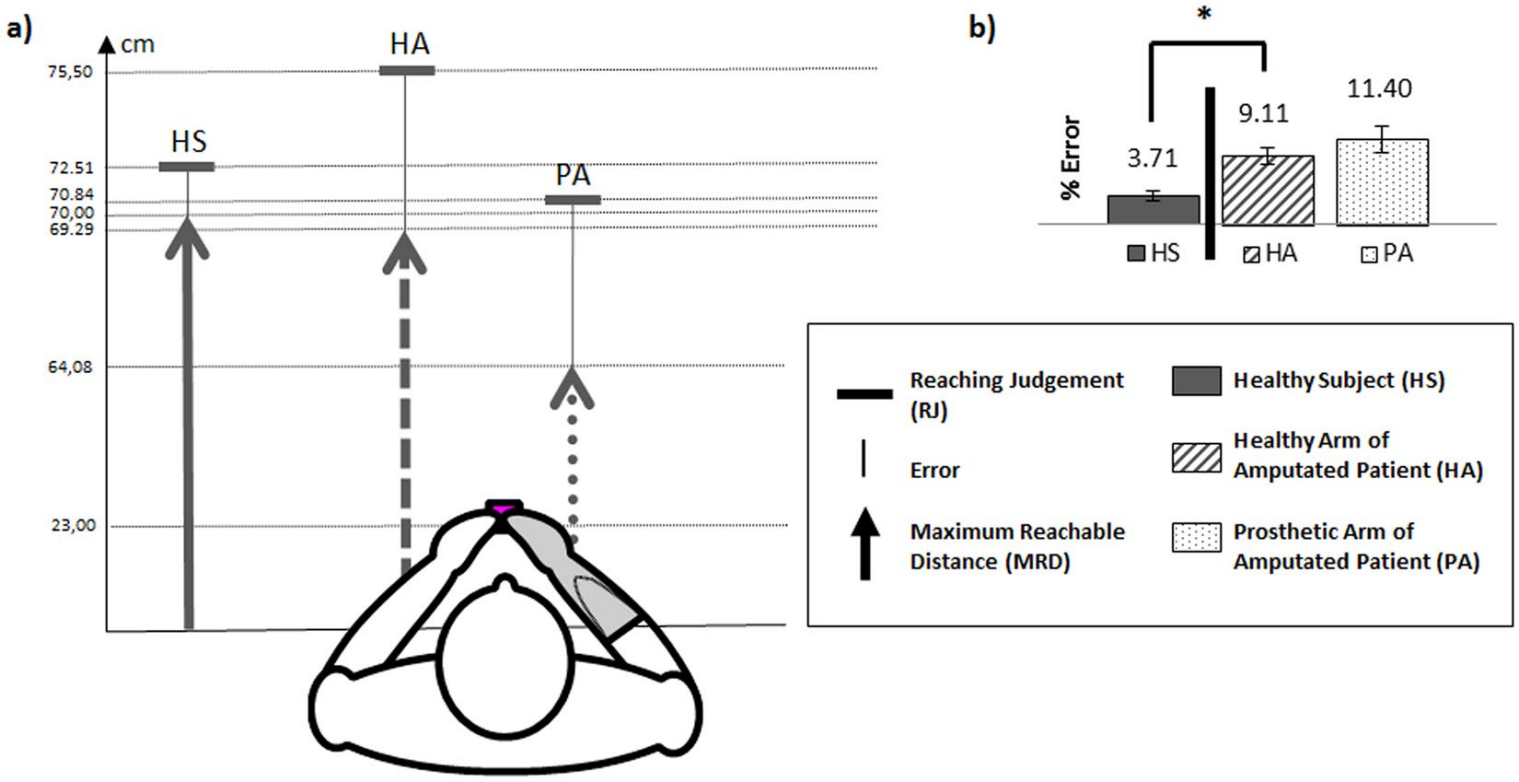
(**a**) MRD (arrow), RJ (perpendicular line) and error, in cm, in healthy subjects and the healthy and prosthetic arms of the patients. Values indicated are the mean obtained over groups and conditions. (**b**) Error of RJ as a % of the MRD, in healthy subjects and the healthy and prosthetic limbs of the patients.

We first compared the RJ to the MRD (as illustrated in Fig. 2a). In the healthy subjects, there was a significant difference between the MRD (70.00 ± 6.23 cm) and the RJ (72.52 ± 6.61 cm; p < 0.01, paired t-test), indicating that the healthy subjects often overestimated their true capacity. This was also found, but with larger values, for the patients for both the healthy arm (MRD = 69.29 ± 5.82 cm; RJ = 75.50 ± 6.46 cm; p < 0.01, paired t-test) and the prosthetic arm (MRD = 64.08 ± 6.71 cm; RJ = 70.84 ± 5.52 cm; p < 0.01, paired t-test).

We then compared the magnitude of the judgement error between the different arms tested, i.e., the difference between RJ and MRD as a percentage of MRD. As shown in Fig. 2b, a Student paired t-test showed no significant differences in the magnitude of error between the patients’arms (healthy arm = 9.11 ± 5.92%, prosthetic arm = 11.40 ± 12.01%; p > 0.05). This result has to be interpreted with reserve because theBayes factor BF_01_ = 2.838 showed anecdotal evidence for the null hypothesis, indicating that more information would be needed to conclude on an equal error with the two arms.However, the magnitude of error made by the patients on their healthy side (9.11 ± 5.92%) was significantly greater than that made by the healthy subjects (3.71 ± 5.44%; p < 0.01, unpaired t-test). These results suggest that patients with amputation overestimate the distance over which they can act more than healthy subjects, both with their healthy and prosthetic arms.

### Reaching judgment is related to the quality of integration

In order to investigate to what extent the evaluation of the capacity of action could vary depending on the degree of integration of the prosthesis, we analysed the relationship between the patient’s’ subjective feelingof integration of the prosthesis, quantified by a score, and their error in reaching judgement.

Patients were asked to answer a questionnaire of prosthetic integration that we designed for this purpose, prior to the experiments. This questionnaire did consider not only the time they spent wearing their prosthesis but also specific aspects of their use. Indeed, the daily time of wear is an objective measurethat enables clinically evaluating the importance of the prosthesis for the patient, (i.e. its usefulness), but it does not allow fine-grained discrimination of the level of integration of the prosthesis nor an understanding of the determinants of this integration. Since a good prosthesis integration should allow a maximal compensation of the handicap, functionally, aesthetically and psychologically^11, 12^, we also evaluated these aspects through a questionnaire.

This questionnaire was subdivided into five sections. The first section was about the quantity of use and the experimenter just asked the patients the number of hours they wore the prosthesis daily (the maximal use was considered to be 12 hours a day or more, noted 10/10). The second section was aboutthe functional quality of use. We extracted 25 items of daily activities (brush their hair or teeth, cook, write, make a call, etc.) from the questionnaire commonlyused in the clinics “Orthotics and Prosthetics Users’ Survey”. Patients were asked to answer from 4 (“really easily”) to 0 (“nearly impossible”) how simple it was for them to use their prosthesis. The third section was about the aesthetic use. We asked patients to judge from 0 (not important) to 10 (really important) how important it was for them to wear their prosthesis for an aesthetic use in 8 contexts of the social life (from “alone at home” to “particular occasions as job interview”). The two last sections asked patients to evaluate from 0 (“not at all”) to 10 (“very much”) how did they think they had appropriated their prosthesis and the extent to which their prosthesis has become indispensable for them (Table 2).

**Table 2:** Integration score.

	Quantity of use		Fonctional use		Aesthetic use		Psychological use		TOTAL
	Hours per day	/10	OPUS	/10	Questionnaire	/10	Appropriation	Indispensability	/10
P1	2	1.7	26	2.6	2	0.6	1.0	1.0	1.5
P2	6	5.0	24	2.4	12	3.8	7.0	6.5	4.6
P3	12	10.0	29	2.9	31	9.7	10.0	10.0	8.5
P4	12	10.0	50	5.0	19	5.9	10.0	9.0	8.1
P5	6	5.0	28	2.8	29	9.1	8.0	7.0	5.9
P6	12	10.0	43	4.3	1	0.3	10.0	10.0	6.9
P7	10	8.3	5	0.5	2	0.6	6.5	8.0	5.0
P8	4	3.3	27	2.7	2	0.6	6.0	6.0	3.2
P9	12	10.0	41	4.1	5	1.6	10.0	8.0	6.9
P10	12	10.0	25	2.5	0	0.0	10.0	10.0	6.5
P11	12	10.0	34	3.4	15	4.7	10.0	8.5	7.5
P12	12	10.0	71	7.1	32	10.0	10.0	10.0	9.4

Weighting: Total = 4 *Quantity of use +2*Functional use +2*Aesthetic use +Subjective appropriation + indispensability.

The data shown in Table 2 illustrates the limitationsin using the quantity of use alone to qualify the integration of prosthesis. Indeed, 7 out of our 12 patients wore their prosthesis more than 12 hours a day, giving similar level. However, they reported very different subjective perception of their prosthesis (in terms of functionality, aesthetic or psychology). This indicates that the total integration score allowed a finer discrimination than the quantity of use alone. Among the obtained results, patient 12 had the highest score. This patient had recently obtained her prosthesis which had immediately become essential for her. She could not imagine not wearing it, even when she was alone at home, and she felt it as an indissociable part of her. In contrast, the patient with the lowest score (P1) had many problems with the healing of his wound, and the fact that his stump was small had complicated fitting. Up to the day of the study, he had never worn it for more than 4 consecutive hours because he found it uncomfortable, and he only wore it for activities which he could not do without it (mainly fishing). He was not concerned by the aesthetic component of the prosthesis and was totally indifferent to the looks of other people when he was not wearing it. Apart from the discomfort aspect, he explained that he did not particularly wish to wear his prosthesis more, and that he got on very well without it.

**Figure 3:**
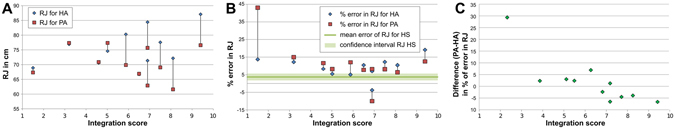
(**A**) Reachability judgement (in cm) for the healthy and prosthetic arms of the patients as a function of the integration score. (**B**) Reachability judgement error (in%) for the healthy and prosthetic arms of the patients as a function of the integration score. Reachability judgement error of healthy subjects (and its confidence interval) is also shown on the plot. (**C**) Difference of the reachability judgement error between the healthy and prosthetic arms as a function of the incorporation score, for 11 patients.

Figure 3A presents the RJ for each patient for both sides plotted as a function of their integration score. Patients with low integration scores tended to estimate similar reaching spaces for the prosthetic and the healthy side while patients with higher interaction scores (over 5.5) tended to judge that they could reach smaller distances with their prosthetic side compared to their healthy one. When considered separately for each side (PA or HA), no correlations was found between the reachability judgement error and the integration score (see Fig. 3B). However, an interesting phenomenon was observed when comparing the percentage of error between the arms: the error with the prosthetic arm was greater than that of the healthy arm for the patients who had a poor feeling of integration whereas it was smaller than that of the healthy arm for the patients who had the greatest subjective feeling of integration (even if the patients remained globally worse at judging their capacity compaired to healthy subjects). This was modelled as the difference of error magnitude in reachability judgement between prosthetic and healthy sides (PA-HA) presented on Fig. 3C, which appeared to be correlated with the subjective level of integration. This relationship was confirmed by Spearman’s rank correlation-coefficient (rho = −0.856, p < 0.001). The correlation between the real MRD and the integration score was tested but found not significant.

Extra analyses were also performed to evaluate, a posteriori, the possible relationship between the reachability judgement error and the quantity of use considered alone and the sum of the three other factors defining integration in a global way (with an equal weight for each: Functional use + Aesthetic use + Psychological use). The result was that the correlation observed with our questionnaire (total score of Table 2) was also found with the wear time considered alone (rho = −0.855, p < 0.001 as Spearman’s rank correlation-coefficient) and with the total score implying only the the three subjective factors without the time (rho = −0.601, p < 0.05).

## Discussion

The main results of this study are three-fold. First, the actual reaching space in amputees was smaller with their prosthesis than with their healthy limb. Yet, they judged that they could reach as far with their prosthesis as with their healthy limb. Second, for their healthy limbs, patients overestimated more the extent of their reaching space than healthy subjects. Finally, their overestimation seems to be smaller with the prosthesis than with the healthy limb when the patients’ score of integration of their prosthesis was high.

Before discussing the perceived ability to act on a nearby space in amputees, it is important to understand their actual motor capacities with their prostheses. In order to study the representation that participants had of their reaching space, we thus first measured their maximum forward reachable distance (MRD). We found that the MRD of the patients’ prosthetic arms was shorter than that of their healthy arms although they were of the same length. A possible explanation is that the prosthesis is fitted over the stump, which is a damaged part of the body and which can lead to physical constraints that limit the possibilities of action (for example, if the stump is short or if the socket prevents full elbow extension). In our study, the reaching capacity of patients was about 5 cm smaller with their prosthesis than with their healthy arm. This shows that the prosthesis only partially restored the physical capacity of the amputated limb to act over a large distance.

Amputated patients overestimated their capacity of action with their prosthesis as much as with their healthy arm (Fig. 2b). This result is in line with Canzoneri and coll.’s^14^ study that showed a restoration of the boundaries of the peripersonal space, which was perceived as farther away from the body when the patients wore their prosthesis. The difference between the two studies is that ours used a sensorimotor task while they used a multisensory task. So we can speculate that both motor and sensory effects resulted from the malleability of body representations that adjusted to the prosthesis, as shown by the increase in perceived arm size with prosthesis^13, 14^. This hypothesis is supported by neurophysiological results showing a reorganisation of bilateral cortical sensorimotor areas after unilateral amputation^34–37^.

These results, argueing in favour of the implicit embodiment of the prosthesis, are encouraging. However, the sensation of having a longer limb while wearing a prosthesis, or the feeling of an extended acting space, are not the goals pursued by patients and the rehabilitation staff. Action planning with a prosthesis requires more than the plasticity of body representations, in particular the adequation of these flexible body representations to the real body shape and capacity to act. Ideally, the error between the actual capacity to act on the environment (MRD) and the perception of the limits of the reaching space (RJ) should be as small as possible. If the prosthesis fails to give patients the same reaching space as their healthy arm (reduced MRD), the body representations should reveal this difference. Estimating to be able to reach farther than what is possible could lead to errors in the anticipation of the optimal trajectory to reach the target.

Interestingly, we observed that not only the representation of the amputated arm was affected by the amputation but also the representation of the healthy arm of the patients. Indeed, the error in reaching judgment was larger for both arms in amputated patients compared to healthy participants. Surprisingly, observing the direction of the error, we found that the patients’ perception is even more optimistic, so to speak, than that of the healthy subjects. In healthy subjects, the standard overestimation in a reaching judgment task is often interpreted as the result of the anticipation of the contribution of trunk movements to reach a more distant area (as in usual non constrained situations) whereas the experimental setup blocked them^38, 39^. Amputated patients may make larger errors because they are accustomed to make larger trunk movements in order to compensate the impaired contribution of the upper-limb to the reaching distance^40, 41^. This might explain the larger error they made for the prosthetic arm. The overestimated reaching distance by the patients on the healthy side is more surprising. Several studies on unilateral impairments, particularly on lower limb amputees wearing a prosthesis, have pointed out some modifications of movement coordination on the healthy side possibly because of body compensatory mechanisms tending to ensure symmetrical actions^42^. Such a large modification of the body as a limb amputation alter the general perception that patients have of the action capacity of their body as a whole because a loss of function of a limb requires a whole-body modification of action strategies, whether wearing or not a prosthesis^43–45^.

While the bilateral overestimation found on the reaching judgment can be the result of a global effect of amputation on body representation, it can also be the result of two different processes on each arm. In favour of this interpretation, we observed that the larger overestimation they made was either for their healthy limb or for their prosthetic limb depended on the quality of the subjective integration of the prosthesis. Indeed, patients with low integration overestimated more with their prostheses than with their healthy limb (i.e., they falsely estimated that they could act with their prosthesis as far as with their healthy arm), whereas patients with good integration showed the reverse pattern (i.e., the amount of error was smaller on the side of the prosthesis than on the healthy side), as shown in Fig. 3C. Nontheless, this result can be appreciated in several ways: it could be that the more the patient felt he had integrated the prosthesis, the more precise his RJ was compared to the error on the healthy arm; or it could be that the more precise he was when judging the action’s capacity of his prosthetic arm (in comparison with his healthy arm) the stronger his subjective feeling of integration is.

This correlation between the extent of the relative error for the prosthetic side and the integration score might be explained by the familiarity and expertise gained from good integration of the prosthesis. Patients who wore their prosthesis a lot (because it allowed them to act more, or because they needed it to look normal or because it gave them the feeling of being complete) seemed more aware of its limitations and did not expect from it to be a perfect substitute of their lost limb. Furthermore, the use of a prosthesis requires more visual control to compensate for the absence of natural proprioception and tactile feedback than the use of the healthy limb, leading to a constant visuomotor remapping of their environment relatively to their prosthesis during action^41, 46^. This can explain their more precise reachability judgement on their prosthetized side in a task involving the visual evaluation of space from a body reference frame. Nonetheless, the overestimation remained bigger than what is observed in healthy subjects suggesting a global overestimation effect.

The questionnaire, revealed not only how much the prosthesis was used but also how the patients experienced it. One may then speculate that these more subjective components of their integration score resulted from the more accurate representation of the reaching distance, and thus of the prosthetic limb metrics. Yet, further experiments are needed to explore this subjective dimension in a larger number of patients. Also, it is possible that the observed correlation were partly influenced by the chosen weights (even if correlations were still found for a “balanced” integration score -ie with equal weights- calculated without the quantity of use). Therefore a deeper analysis of individual factors within the correlation with action representation could be enlightening.

In conclusion, amputation and the wearing of a prosthesis modifies the body representation on both the prosthetic and healthy sides. The subjective quality of the integration of the prosthesis, including the feeling of embodiment, appears to influence these modifications. It seems that good integration of prostheses depends as much on their use, sparing the healthy limb as much as possible, as on the knowledge of their imperfections and limitations.

## Method

### Participants

Thirty healthy subjects of the Institute of Intelligent Systems and Robotics (25 males and 5 females, aged from 23 to 68 years, mean 41 years) were included. A convenience sample of 12 patients with upper limb amputation (10 males and 2 females aged from 22 to 61 years, mean 41 years) was included between the 19^th^ of March 2015 and the 15^th^ of September 2015 at the Regional Rehabilitation Institute in Nancy. The only inclusion criteria were amputation at the level of the forearm, in possession of a myoelectric prosthesis and normal, or corrected to normal, vision. There were no exclusion criteria, thus the time elapsed since amputation as well as the cause and complexity of the prosthetic fitting varied greatly across patients (Table 1). The protocol was approved by our local ethics committee (Conseild’évaluationéthique pour les recherchesen santé de Paris Descartes N° IRB: 20151900001072) and the study was performed in accordance with the Declaration of Helsinki. All the subjects gave written informed consent prior to participating.

### Experimental protocol

Both the healthy and prosthetic arms (limb with amputation wearing the prosthesis) of the patients were tested in a randomised order. For 9 of the 12 patients (75%), the healthy arm was the non-dominant arm. In order to have an equivalent proportion, 22 healthy subjects (73%) of the 30 carried out the test with their non-dominant arm while the other 9 used their dominant arm.

**Figure 4:**
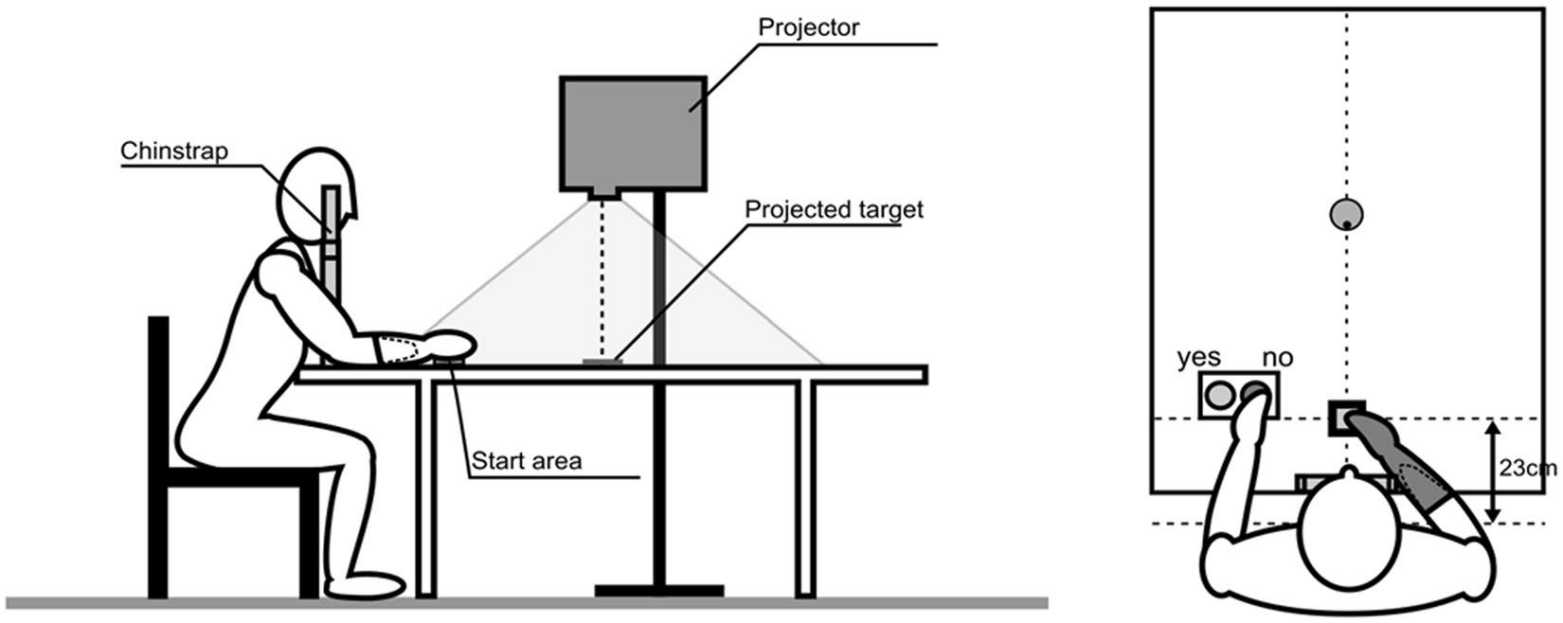
Experimental set-up The positions of the targets and associated responses were recorded by a computer.

The experimental set-up consisted of a table (1 m wide and 1.20 m deep) which was visually isolated from the external environment by a black cloth. A projector was placed on a tripod on the right side of the table and projected computer-generated images on the centre of the table (Fig. 4).

To limit trunk and shoulder motion, the chin was positioned on a chin-rest fixed to the table and the seat was adjusted so that the patient’s belly contacted the edge of the table. Then, for each condition (healthy arm, and amputated arm with prosthesis), the maximal reachable distance (MRD) was measured by asking the patient to extend the arm as much as possible to touch the furthest point on the table in front of him/her, in the sagittal plane. This was carried out with the eyes shut so as not to give any visual reference for the remaining of the experiment. Subjects also wore headset with integrated hearing protections.

Second, the subjects were asked to judge their capacity to reach the targets projected onto the table with the tested arm, without actually moving the arm. Throughout the whole experiment, the hand was positioned in front of them at 23 cm from the body in the sagittal axis. The visual targets (green circles of 10 mm diameter) were presented at 25 positions along the subject’s sagittal axis, on a black background. These 25 positions covered a distance of +9.6 to −9.6 cm (in steps of 8 mm) from the maximal reaching distance measured for the tested limb^33^. Each position was tested 5 times in a randomised order. For each trial, the subjects were expressing their reachability judgement by pressing on a green button to indicate “yes, I can reach this target”, or on a red button to indicate “no, I cannot reach this target”. The following target appeared after a randomised delay of 500 to 1000 ms.

### The positions of the targets and associated responses were recorded by a computer

The perceived limit of the reachable space for each participant in each condition was determined by an individual logistic regression model for binomial distribution^47^, calculated on the basis of the percentage of “yes, I can reach this target” responses out of the total number of responses for each of the 25 target positions. The intersection of this curve (p = 0.5) denoted the reaching limit or reachability judgment (RJ) for each condition for each subject. The percentage of error of RJ by reference to MRD was calculated.

We considered values which were greater than 2.173 times the standard deviation to be aberrant and excluded them from the analysis as described in Selst *et al*.^48^ (11 of the 3000 data points, i.e., 0.37%, in the patients with amputation: 4 with the healthy arm and 7 with the prosthetic arm; and 12 out of 3750, i.e., 0.3%, in the healthy subjects). The data analysis was carried out using Microsoft® Excel and Matlab® software. The mean values and confidence interval obtained in healthy subjects were calculated and used for analysis of the results in individual patients. The statistical comparison of the values (RJ, MRD and % of error) obtained between three conditions (HS, HA, PA) was performed with Student’s t test. A Bonferroni correction for multiple comparisons was used, reducing the limit for a significant p value to 0.02. When the t test did not show significant difference, further analysis was performed with Bayesian t test statistics (jasp software https://jasp-stats.org)^49^.

Two patients (P1 and P3) overestimated their capacity of action to such an extent that they judged that they were capable of reaching all the targets (up to over 9.6 cm of their MRD). We thus repeated the test in P1 with targets positioned at a greater distance in order to calculate his RJ, however P3 could not repeat the test, so his data were removed from the analysis.

### Interviews

At the end of the experiment, the patients were asked about the causes and circumstances of their amputation and the modalities of their prosthetic fitting. They had then to reply to questions about the integration of their prosthesis. Clinicians often evaluate prosthetic integration on the basis of the time spent wearing the prosthesis but what they qualified as a “good integration” goes beyond that quantitative measure. A “good prosthesis” restores not only the ability to act, but also the social image and the feeling of body integrity^11, 12^. We therefore designed a scale of prosthetic integration following numerous preliminary interviewswith patients and clinicians. The selection of the factors included has been established discussing with experts, prior to experiments and was not driven by a posterior experimental evidences. Our scale gives equal weight to three previously listed factors: functionality, social image and phenomenology. The evaluation of the compensation of the functional disability is the only one frequently made by the rehabilitation staff using for this purpose the OPUS questionnaire^50^. In this questionnaire, patients have to rate on a numerical scale (from 0 to 4, maximal score = 100, calculated out of 10) if they wore their prosthesis to carry out specific activities of daily life and if they do, how simple it was for them. Here we used 25 out of the 28 items, removing the questions that did not apply to our patients (e.g. they were not allowed to take a shower or to drive with their prosthesis). In the absence of standard questionnaires about the compensation of the aesthetic handicap, we designed one with a structure similar to the OPUS. The patients were asked to rate (from 0 to 4, maximal score = 32, calculated out of 10) how much they needed to hide their amputation by wearing the prosthesis in 8 different daily contexts: alone at home, with their partner, with persons living with them, with the extended family, their friends, at work and in the street and for particular occasions (evenings out, job interview etc.). The last questionnaire concerns the feeling of body integrity, which can be analysed into two components, one active and one passive. First, the feeling of appropriation describes the active process by which the patients make their prosthesis a constitutive part of themselves (some of them tattoo it or they wear jewelry around it). The feeling of indispensability describes how they experience the prosthesis as an indissociable part of their body (e.g., without it, they feel that something is missing). We asked patients to evaluate these two factors by means of two numerical scales (from 0 to 10). The questionnaire finally took into account the number of hours the prosthesis was worn per day (maximal score ≥12, calculated out of 10). Each criterion was calculated out of 10 and so was the final score (100 points = 10/10).A total “integration score” was then calculated from all these subjective evaluations (Total = 4*Quantity of use + 2*Functional use + 2*Aesthetic use + Appropriation + Indispensability). We used arbitrary weights, motivated by the desire of having an equal importance for the 3 factors (functional, aesthetic and psychological divided itself in two sub-aspects) and a stronger weight for the time of wearing (the most reliable quantified index).

Spearman’s rank correlation analysis was used to investigate the relationships between the explicit integration score and implicit reaching capabilities. Extra analyses were performed a posteriori (Spearman’s rank correlation) to observe separately the correlations with the quantity of use (consequence of the level of integration), and with the determinants of this integration (the reasons for which patients wear their protsthesis a variable time during the day).
